# UvKmt6-mediated H3K27 trimethylation is required for development, pathogenicity, and stress response in *Ustilaginoidea virens*

**DOI:** 10.1080/21505594.2021.2008150

**Published:** 2021-12-11

**Authors:** Shuai Meng, Zhiquan Liu, Huanbin Shi, Zhongling Wu, Jiehua Qiu, Hui Wen, Fucheng Lin, Zeng Tao, Chaoxi Luo, Yanjun Kou

**Affiliations:** a State Key Laboratory of Rice Biology, China National Rice Research Institute, Hangzhou, China; b Hubei Key Laboratory of Plant Pathology, and College of Plant Science and Technology, Huazhong Agricultural University, Wuhan, China; c State Key Laboratory of Rice Biology, Institute of Biotechnology, Zhejiang University, Hangzhou, China; d State Key Laboratory for Managing Biotic and Chemical Threats to the Quality and Safety of Agro-products, Institute of Plant Protection and Microbiology, Zhejiang Academy of Agricultural Sciences, Hangzhou, China

**Keywords:** *Ustilaginoidea virens*, virulence, PRC2, Kmt6, epigenetic regulation

## Abstract

Polycomb repressive complex 2 (PRC2) is responsible for the trimethylation of lysine 27 of histone H3 (H3K27me3)-mediated transcriptional silencing. At present, its biological roles in the devastating rice pathogenic fungus *Ustilaginoidea virens* remain unclear. In this study, we analyzed the function of a putative PRC2 catalytic subunit UvKmt6. The results showed that disruption of *UvKMT6* resulted in reduced growth, conidiation and pathogenicity in *U. virens*. Furthermore, UvKmt6 is essential for establishment of H3K27me3 modification, which covers 321 genes in the genome. Deletion of *UvKMT6* led to transcriptional derepression of 629 genes, 140 of which were occupied with H3K27me3 modification. Consistent with RNA-seq and ChIP-seq analysis, UvKmt6 was further confirmed to participate in the transcriptional repression of genes encoding effectors and genes associated with secondary metabolites production, such as *PKS*s, *NRPS*s and *Cytochrome P450s*. Notably, we found that UvKmt6 is involved in transcriptional repression of oxidative, osmotic, cell wall and nutrient starvation stresses response-related genes. From the perspective of gene expression and phenotype, in addition to the relatively conservative role in fungal development, virulence and production of secondary metabolites, we further reported that UvKmt6-mdediated H3K27me3 plays a critical role in the response to various stresses in *U. virens*.

## Introduction

Epigenetic regulation is dependent on modifications of genomic DNA or histone, but not on the changes of DNA sequences [1]. It is known that the lysine methylation of histone H3 plays an important role in epigenetic regulation. In general, di-/tri- methylation of H3 lysine 4 and H3 lysine 36 are responsible for transcriptional activation, while di-methylation of H3 lysine 9 and tri-methylation of H3 lysine 27 (H3K27me3) are involved in transcriptional silencing [2]. Among these modifications, H3K27me3 is conserved from fungi to higher organisms such as *Arabidopsis, Drosophila* and mammals [3]. The H3K27me3 modification was first found in *Drosophila* and mediated by polycomb repressive complex 2 (PRC2) [4]. The PRC2 complex consists of core subunits Ezh (Kmt6), Su(z)12, Esc (Eed) and additional subunits RbAp48/Nurf55 (P55), among which Ezh is a key catalytic subunit of PRC2 [5]. Subsequent studies have shown that Ezh (Kmt6)-mediated H3K27me3 modification is involved in the development and differentiation of various organisms. For example, Ezh2, homologue of Ezh, is up-regulated in many cancer cells and associated with tumorigenesis of breast, pancreatic, colorectal and endometrial cancer [6,7]. In addition, Ezh2 is essential for maintaining intestine integrity and caudal fin regeneration in zebrafish [8] and early gastrulation development in mouse [9]. In model plant *Arabidopsis*, accumulation of H3K27me3 modification on *Flowering Locus C* (*FLC*) regulates developmental transition induced by vernalization [10].

H3K27me3 modification on chromatin plays distinct roles in regulation of differentiation and development in filamentous fungi. In *Neurospora crassa*, H3K27me3 modification mediated by PRC2 covers 6.8% of genome. Deletion of *SET7* (*Ezh/KMT6* homologues) caused loss of H3K27me3, but did not result in defects of growth and sexual development in *N. crassa* [3]. Similarly, absence of H3K27me3 modification does not lead to obvious phenotypic changes and gene transcriptional activation in pathogenic fungus *Zymoseptoria tritici*, although it reduces the loss of accessory chromosomes [11]. It was further found that H3K27me3 is involved in spatiotemporal expression regulation of virulence-related *effectors* in *Z. tritici* [12]. In contrast, in the wheat head blight fungus *Fusarium graminearum*, growth and the production of various metabolites are regulated by Kmt6-mediated repression [13]. Knockdown of *KMT6* also induced derepression of secondary metabolites gene clusters in the rice bakanae pathogen *Fusarium fujikuroi*, which led to increased synthesis of secondary metabolites, such as beauvericin [14]. Furthermore, Kmt6-mediated H3K27me3 modification is required for fungal growth and development in *F. fujikuroi* [14]. In addition to plant pathogens, in a beneficial endophyte *Epichloe festucase*, H3K27me3 plays an important roles in building symbiotic interaction with *Lolium perenne* by regulating specific expression of alkaloid biosynthesis-related genes [15]. In the rice blast fungus *Magnaporthe oryzae*, MoKmt6 catalyzes H3K27me3 and is required for growth, conidiation, pathogenicity and orchestration of *effector*s transcription [16,17]. A recent report in the fungus *Podospora anserina* revealed that loss of Kmt6 also resulted in severe defects in growth, and differentiation by causing genome-wide loss of H3K27me3 modification [18]. Therefore, PRC2-mediated H3K27me3 modification in fungi is conservative, but has multiple functions, which is worthy of further study.

*Ustilaginoidea virens*, the causal agent of the rice false smut disease, is an economically important fungal pathogen posing threats to rice production [19,20]. The infection of *U. virens* not only reduces the quality and yield of rice, but also contaminates the rice seeds and straws with toxins, such as ustiloxins and ustilaginoidins [21–23]. Recently, a great progress has been made in depicting infection process of *U. virens*. Briefly, at the booting stage, the *U. virens* conidia germinate and enter into the rice spikelet through the space between lemma and palea to infect stamens [24]. Subsequently, invasive hyphae spread into stigmas and fill the floral organs, finally develop into false smut balls, which are typical and visible symptom of the rice false smut disease [25]. In order to complete the infection, *U. virens* needs to adapt to inevitable stresses from host and environment in variety of ways, such as secreting a lot of effectors to modulate rice immunity [26–29] or regulations of stress response-related pathways [30–33]. Although a great progress has been made in the research of rice false smut fungus, the infection mechanism of *U. virens* is still largely unclear. Revealing its pathogenic mechanism is of great significance for formulating effective control strategies.

In this study, we set out to investigate the biological roles of H3K27me3 modification in *U. virens*. By disruption of *UvKMT6*, we found that it is essential for H3K27me3 modification and required for growth, consolidation and virulence. Furthermore, in combination with ChIP-seq, RNA-seq, and phenotypic analysis, we revealed that H3K27me3-mediated transcriptional repression kept tight correlation with *effector* transcription, secondary metabolism, and stress responses in *U. virens*.

## Results

### *Identification of* UvKMT6 *in* U. virens

**Figure 1.**
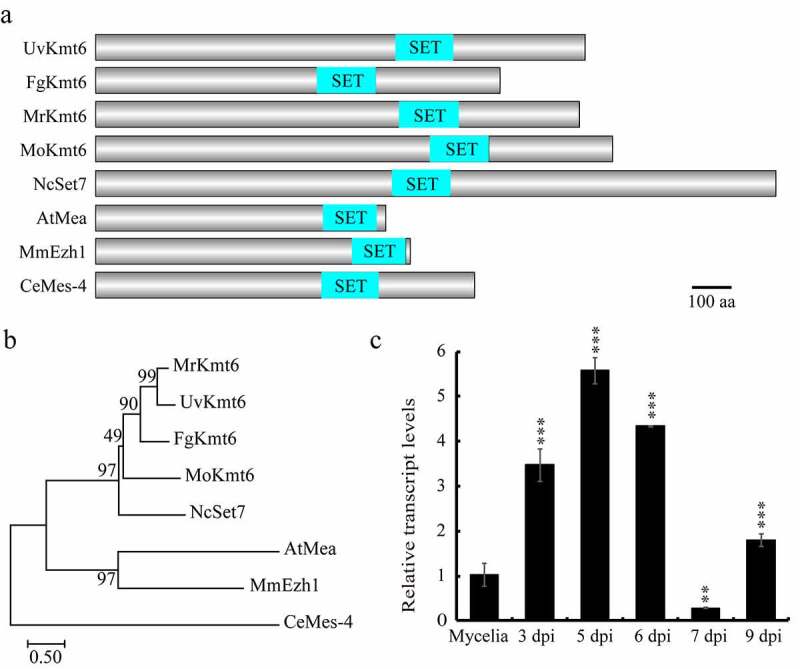
**Identification of *UvKMT6* in *U. virens***. (a) The Kmt6 homologous proteins share a conserved SET domain. The Kmt6 homologous analyzed included *Ustilaginoidea virens* Kmt6 (KDB18066.1), *Fusarium graminearum* Kmt6 (XP_011316633.1), *Metarhizium robertsii* Kmt6 (XP_007816866.1), *Magnaporthe oryzae* Kmt6 (XP_003718975), *Neurospora crassa* Set7 (XP_965043.2), *Arabidopsis thaliana* Mea (NP_563658.1), *Mus musculus* Ezh1 (XP_006532241.1) and *Caenorhabditis elegans* Mes-4 (NP_506333.1). Bar, 100 amino acids. (b) Phylogenetic analysis of Kmt6 homologous from various organisms. (c) The relative expression level of *UvKMT6* gene in *U. virens*. Using the *β-tubulin* gene as internal control, the expression level of *UvKMT6* during infection stages was calibrated with that of vegetative growth stage. The data represent the mean ± SD from three independent replicates. Asterisks represent significant difference at P value ˂ 0.001

By BLASTp with *F. graminearum* Kmt6 (XP_011316633.1) protein sequence as a query, Kmt6 homologues including *U. virens* Kmt6 (KDB18066.1), *Metarhizium robertsii* Kmt6 (XP_007816866.1), *M. oryzae* Kmt6 (XP_003718975), *N. crassa* Set7 (XP_965043.2), *A. thaliana* Mea (NP_563658.1), *Mus musculus* Ezh1 (XP_006532241.1) and *Caenorhabditis elegans* Mes-4 (NP_506333.1) were identified. All of these Kmt6 homologues contain a SET domain predicted by the SMART tool (http://smart.embl-heidelberg.de) (Figure 1a). Sequence alignment of eight Kmt6 homologues showed that Kmt6 homologues displayed high sequence similarity, ranging from 47% (*M. musculus*) to 75% (*M. robertsii*) (Figure S1). Furthermore, a phylogenetic tree of Kmt6 homologues constructed by the MEGA 7.0 software showed that UvKmt6 was closely related to *M. robertsii* Kmt6 (Figure 1b). These results indicated that Kmt6 is highly conserved in different fungi, and may play an important role in *U. virens* as other fungi (Figure 1b).

Next, expression level of *UvKMT6* at different infection stages including 3, 5, 6, 7 and 9 dpi (days post inoculation) was monitored by qRT-PCR (quantitative Real-time PCR). The results showed that the expression level of *UvKMT6* during the infection process increased more than two folds comparing with that of mycelial stage except 7 dpi (Figure 1c), implicating a possible role of *UvKMT6* during infection stage in *U. virens*.

### *Targeted gene deletion and complementation of* UvKMT6

**Figure 2.**
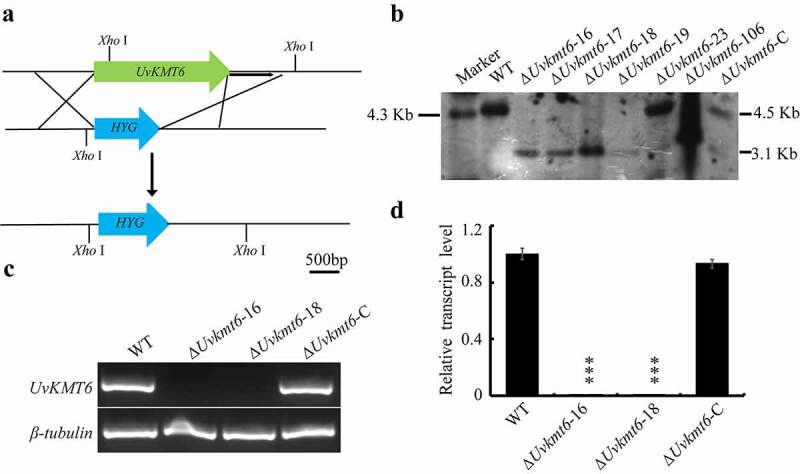
**Construction and verification of *UvKMT6* deletion mutant**. (a) Scheme of *UvKMT6* disruption strategy. *HYG, hygromycin resistance gene cassette*. (b) Southern blot analysis of the WT, *UvKMT6* knockout mutants and complemented strain Δ*Uvkmt6*-C. Genomic DNA of the indicated strains was digested by *Xho*I and subject to Southern blotting with a probe located downstream of the *UvKMT6* coding region as shown in the Figure 1a. In the correct *UvKMT6* knockout mutants, a 4.5 Kb band in WT was shift to 3.1 Kb. (c) Verification of *UvKMT6* deletion mutants by RT-PCR analysis. Compared with the obvious *UvKMT6* expression in WT and Δ*Uvkmt6*-C, no visible band was detected in the Δ*Uvkmt6*-6 and Δ*Uvkmt6*-18 mutants. Similar results were obtained from two repeats. (d) The expression levels of *UvKMT6* was determined by qRT-PCR assay. The expression level of *UvKMT6* gene in the complemented strain Δ*Uvkmt6*-C is comparable to that of the WT strain. Asterisks represent statistically significant differences at P value ˂ 0.001 (***) or P value ˂ 0.005 (**)

**Figure 3.**
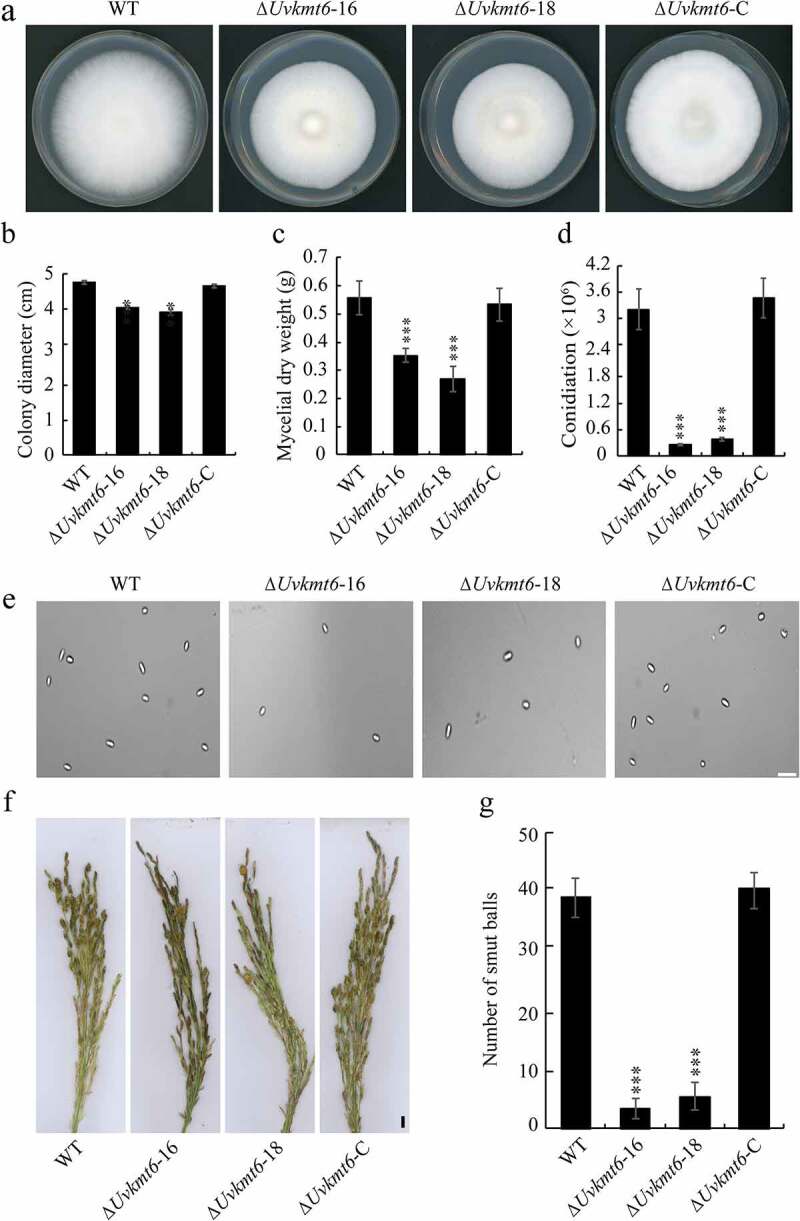
**Disruption of *UvKMT6* resulted in reduced growth, conidiation, and pathogenicity in *U. virens***. (a) Colonies of the WT, Δ*Uvkmt6* and complemented strains. Mycelial plugs of the WT, Δ*Uvkmt6* and complemented strains were cultured on PSA plates for 14 d, and then photographed. (b) The colony diameters of the indicated strains. (c) UvKmt6 plays important roles in hyphal growth in *U. virens*. Mycelia of indicated strains cultured in PS media for 7 d were harvested and weighed after drying. (d and e) Knockout of *UvKMT6* gene resulted in decreased conidation in PS medium. The mycelial plugs of the WT, Δ*Uvkmt6* and Δ*Uvkmt6*-C strains were cultured in equal volume of liquid PS medium. At 7 dpi, conidiation were measured by determining conidial concentration in the PS liquid medium. Conidia in the liquid media were counted with hemocytometer and photographed under microscope. Bar = 5 μm. (f and g) The number of smut balls formed on the inoculated rice panicles with the Δ*Uvkmt6* strain was less than those of the WT and complemented strains. Bar, 1 cm. Values represent the mean ± SD from three independent repeats. * or *** indicate P value <0.01 or <0.001 compared to the WT

To investigate biological roles of *UvKMT6* in *U. virens, UvKMT6* deletion mutants were constructed based on the principle of homologous recombination (Figure 2a). Then, Southern blotting and qRT-PCR assays were used to verify that *UvKMT6* gene was replaced by the *hygromycin resistance gene cassette*. In the deletion mutants, the 4.5 Kb-band in WT shifted to 3.3 Kb, suggesting that Δ*Uvkmt6*-16, 17, 18 and −19 were correct deletion mutants without ectopic insertions (Figure 2b). In addition, we confirmed that the transcriptional expression of *UvKMT6* was undetectable in the Δ*Uvkmt6*-16 and −18 strains (Figure 2c). Therefore, Δ*Uvkmt6*-16 and Δ*Uvkmt6*-18 mutants were chosen for following characterization. To verify that the phenotypic difference (Figure 3a) between the ∆*Uvkmt6* and WT strains was caused by loss of *UvKMT6*, the complementation strains Δ*Uvkmt6*-C were created by introducing the corresponding WT *UvKMT6* locus into the Δ*Uvkmt6*-16 mutant with the ATMT (*Agrobacterium tumefaciens-*mediated transformation) method. Southern blotting and qRT-PCR assays of resultant Δ*Uvkmt6*-C strain showed that *UvKMT6* has been introduced into the deletion mutant and was transcriptionally expressed normally (Figure 2b-d). In addition, the phenotypes of Δ*Uvkmt6*-C strains were similar to those of the WT strain (Figure 3a), suggesting that *UvKMT6* functionally complemented the Δ*Uvkmt6* phenotype.

### UvKMT6 *is required for growth, conidiation, and virulence in* U. virens

Because the colonies of ∆*Uvkmt6* mutants appeared to be smaller than that of the WT strain, hyphal growth was evaluated by inoculating mycelial plugs of the WT, Δ*Uvkmt6*-16, −18 and Δ*Uvkmt6*-C strains on the PSA (potato sucrose agar medium) plates. As the Figure 3a and Figure S2 displayed, the colony diameter of Δ*Uvkmt6*-16 and −18 were slightly reduced than WT during the cultured process, indicating that UvKmt6 facilitates hyphal growth. This role was further confirmed by measuring mycelial weight in liquid cultures. When cultured in liquid PS (potato sucrose medium) culture for 7 d (days), mycelial dry weights of the Δ*Uvkmt6*-16 and −18 strains were also less than those of WT (Figure 3c). In contrast, the vegetative growth was rescued in the Δ*Uvkmt6*-C strain (Figure 3), suggesting that UvKmt6 plays important roles in mycelial growth in *U. virens.*

Kmt6 homologues were reported to be involved in asexual development in *M. oryzae* and *F. graminearum* [13,16], but not in *N. crassa* [3]. To determine whether UvKmt6 was required for conidiation in *U. virens*, the same amount of mycelial plugs of the WT, Δ*Uvkmt6* and Δ*Uvkmt6*-C strains were cultured in equal volume of liquid PS medium. After culture for 7 days in a shaker, conidiation were measured by determining conidial concentration in the PS liquid medium. Conidia in the liquid media were counted with hemocytometer and photographed under microscope. The results showed that deletion of *UvKMT6* caused a significant reduction (approximate 90%) in conidiation compared with those of WT and Δ*Uvkmt6*-C strains (Figure 3d, e), suggesting that *UvKMT6* is involved in asexual development in *U. virens*.

To examine whether UvKmt6 is required for the pathogenicity of *U. virens*, the mixtures of mycelia and conidia of WT, Δ*Uvkmt6*-16, −18 and complemented strains were injected into booting-stage panicles of susceptible rice cultivar Wanxian 98 (*Oryza sativa* L. *indica*), respectively. Three weeks after inoculation, it was found that the Δ*Uvkmt6*-16 and −18 developed less smut balls on inoculated panicles than WT (Figure 3f and g). In contrast, the reintroduction of *UvKMT6* restored the virulence to the WT level. These results suggested that UvKmt6 contributes to fungal virulence in *U. virens*.

### UvKmt6 is essential for the establishment of histone modification H3K27me3

**Figure 4.**
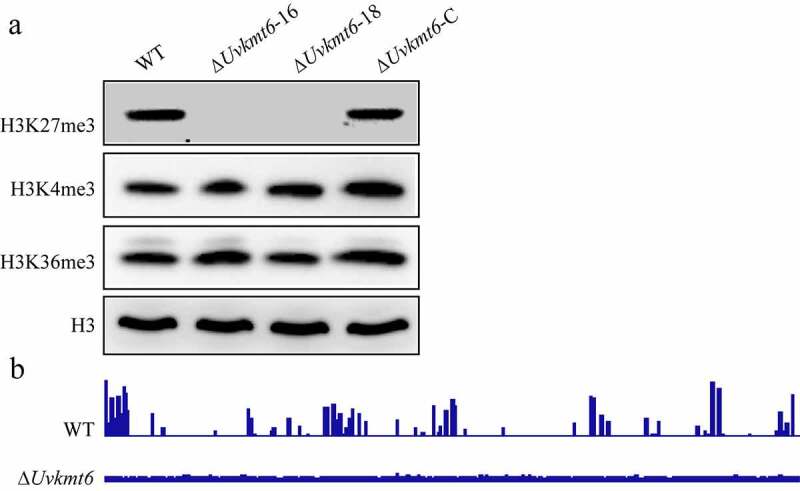
**UvKmt6 is essential for establishing H3K27me3 modification in *U. virens***. (a) Deletion of *UvKMT6* resulted in loss of H3K27me3 modification. Total nuclear proteins of WT, Δ*Uvkmt6* and complemented strain Δ*Uvkmt6*-C were isolated and separated by 15% SDS-PAGE (Sodium dodecylsulphate polyacrylamide gel electrophoresis) gel electrophoresis. Protein blots with specific H3, H3K27me3, H3K4me3 and H3K36me3 antibodies indicated that UvKmt6 is specifically involved in H3K27me3 modification. (b) The distribution of H3K27me3 modification in the WT and Δ*Uvkmt6* strains. H3K27me3-marked genomic sequences in the WT and Δ*Uvkmt6* strains were immunoprecipitated with H3K27me3 antibodies, and then sequenced. There was enrichment of H3K27me3 modification in the WT, but not in the Δ*Uvkmt6* strain. Two repeated biological experiments were carried out with similar results

To explore whether UvKmt6 is indeed required for H3K27me3 modification, nucleic proteins of WT, Δ*Uvkmt6*-16, −18 and complemented strains were extracted and subjected to immunoblot with specific H3K27me3 antibodies. In the Δ*Uvkmt6*-16 and −18 mutants, the levels of H3K27me3 modification were almost undetectable compared to obvious bands in the WT and complemented strain Δ*Uvkmt6*-C (Figure 4a). In contrast, trimethylation of H3K36 and H3K4 modification, which are required for activation of transcription, did not show obvious changes after disruption of *UvKMT6* (Figure 4a). These results indicated that UvKmt6 is specifically required for H3K27me3 modification.

To further analyze the deposition of H3K27me3 on the chromatin, ChIP-seq assay was carried out with WT and Δ*Uvkmt6*-16 strains. In the WT, H3K27me3 modification showed high enrichment and marked specific chromosomal regions (Figure 4b and supplemental table S2). In contrast, H3K27me3 peaks were nearly undetectable in the Δ*Uvkmt6* mutant, which was consistent with the aforementioned immunoblotting results. These results further indicated that UvKmt6 is essential for H3K27me3 modification in *U. virens*.

### UvKmt6-mediated H3K27me3 plays a vital role in transcriptional repression

**Figure 5.**
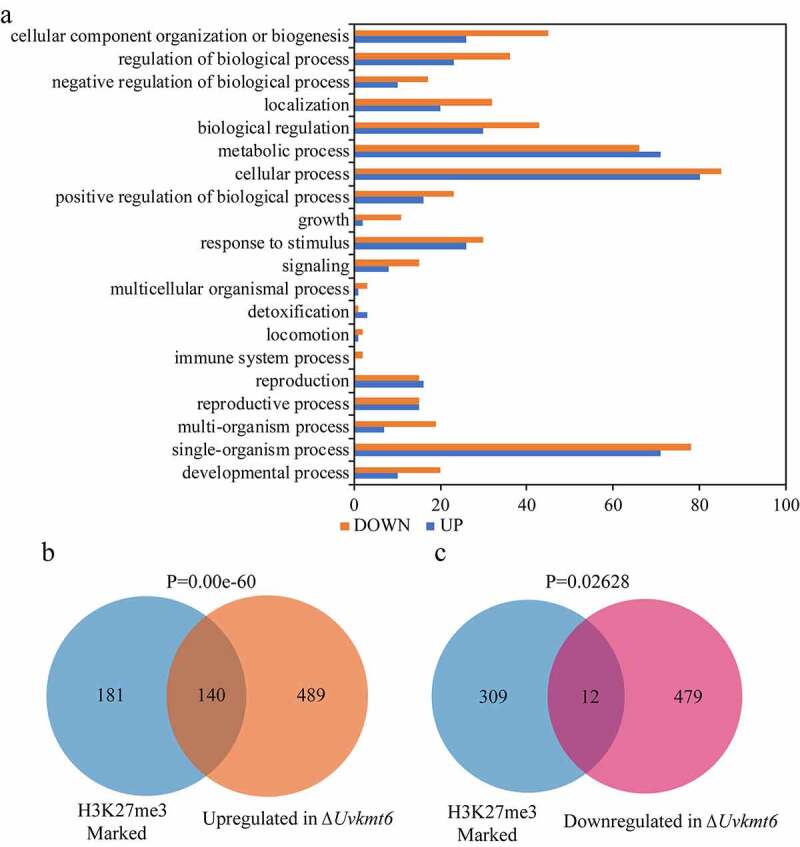
**UvKmt6-mediated H3K27me3 is mainly associated with transcriptional repression**. (a) Gene Ontology (GO) analysis of differentially expressed genes (DEGs) between the Δ*Uvkmt6* mutant and WT strain. DEGs were significantly enriched in biological processes including cellular development and differentiation process, metabolic process and response to stimuli. (b) Venn diagrams show the overlap between H3K27me3-marked genes and up-regulated genes in the Δ*Uvkmt6* mutant when compared with WT strain. (c) Venn diagrams display the overlap of 12 genes between H3K27me3-marked genes and down-regulated genes in the Δ*Uvkmt6* mutant

It is well known that Kmt6-mediated H3K27me3 modification plays vital roles in gene silencing [5]. To investigate the roles of H3K27me3 in the transcriptional regulation in *U. virens*, RNA-seq analysis was performed using vegetative mycelia of WT and Δ*Uvkmt6* cultured in the liquid PS medium for 7 d. Compared with the WT strain, a total of 1120 genes were differentially expressed in Δ*Uvkmt6* mycelia (adjusted p value < 0.01), with 629 genes up-regulated (log_2_ > 1) and 491 down-regulated (log_2_ < −1). Gene ontology (GO) analysis of differential expression genes (DEGs) showed that DEGs were involved in various important biological processes: cellular process (165/1120), development process (30/1120), positive and negative regulation of biological process (27/1120 and 39/1120), metabolic process (137/1120) and cellular component organization or biogenesis (71/1120), response to stimuli (56/1120) (Figure 5a). These data implied the involvement of UvKmt6-mediated H3K27me3 modification in extensive biological processes in *U. virens*.

To determine the relationship between H3K27me3 enrichment and transcriptional regulation, comparative analysis was performed with three gene sets among H3K27me3-occupancy genes, up- and down-regulated genes in Δ*Uvkmt6*. As shown in Figure 5b and c, the 140 out of 629 up-regulated genes in the Δ*Uvkmt6* mutant were H3K27me3 enriched genes, while only 12 down-regulated genes showed H3K27me3 enriched genes. The other 489 up-regulated genes may be indirectly regulated by H3K27me3 modification. These results suggested that the de-repressed genes in the ∆*Uvkmt6* are highly correlated with the absence of H3K27me3 occupancy in *U. virens*.

### *UvKmt6-mediated H3K27me3 modification regulates transcription of* effectors *in* U. virens

**Figure 6.**
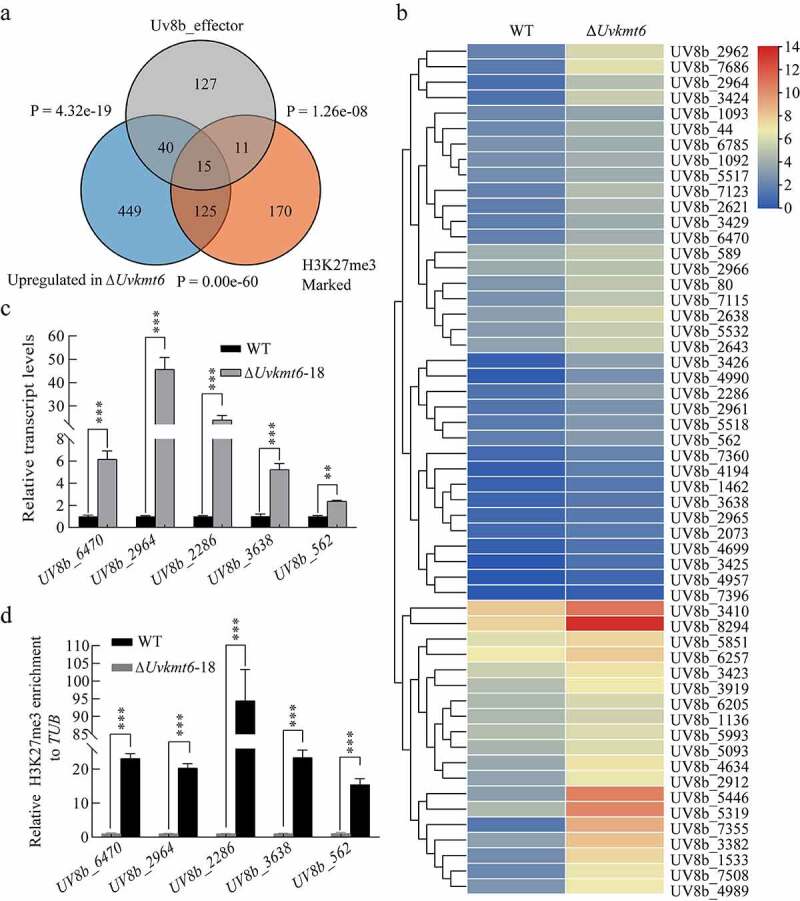
**UvKmt6-mediated H3K27me3 modification suppresses the expression of a large number of *effectors* at vegetative growth stage in *U. virens***. (a) The overlapping between *U. virens* 193 predicted *effectors*, up-regulated genes in Δ*Uvkmt6*, and H3K27me3-marked genes was present by venn diagrams. The association significance of two gene sets was calculated by Fisher’s exact test with P values labeled. (b) Heatmaps showing up-regulated expression levels of 55 *effectors* in Δ*Uvkmt6* compared with those of WT during vegetative growth stage. (c) The relative transcriptional levels of 5 representative *effectors* were determined by qRT-PCR. (d) ChIP-qPCR verified the enrichment of H3K27me3 modification on the chromatin of representative *effectors* in the WT strain. Data represents mean ± SD of three independent replicates. ** or *** represent P value < 0.005 or < 0.001 compared to that of WT

Recently, fungal histone modification H3K27me3 was demonstrated to repress transcription of *effectors*, which are specific expressed during *in planta* growth stage to contribute to pathogenicity [12,15,17]. In *U. virens*, there are at least 193 putative effectors [26]. Among them, transcriptional levels of 55 putative *effectors* are up-regulated during the vegetative growth stage when *UvKMT6* was disrupted (Figure 6a and b). Notably, there were five *effector* genes occupied with H3K27me3, including *Uv8b_6470, Uv8b_2964, Uv8b_2286, Uv8b_3638,* and *Uv8b*_*562*, which have been reported to be involved in suppression of host immunity [26]. Furthermore, qRT-PCR and ChIP-qPCR analysis confirmed that these 5 *effectors* increased their expression for 2.5 to 45 folds (Figure 6c) and decreased their H3K27me3-occupancy for more than 90% (Figure 6d) in the ∆*Uvkmt6* in comparison with those in WT. These results indicated that UvKmt6-mediated H3K27me3 modification participated in the transcriptional repression of *effectors* during vegetative growth stage in *U. virens*.

### Secondary metabolites-related genes are regulated by UvKmt6

**Figure 7.**
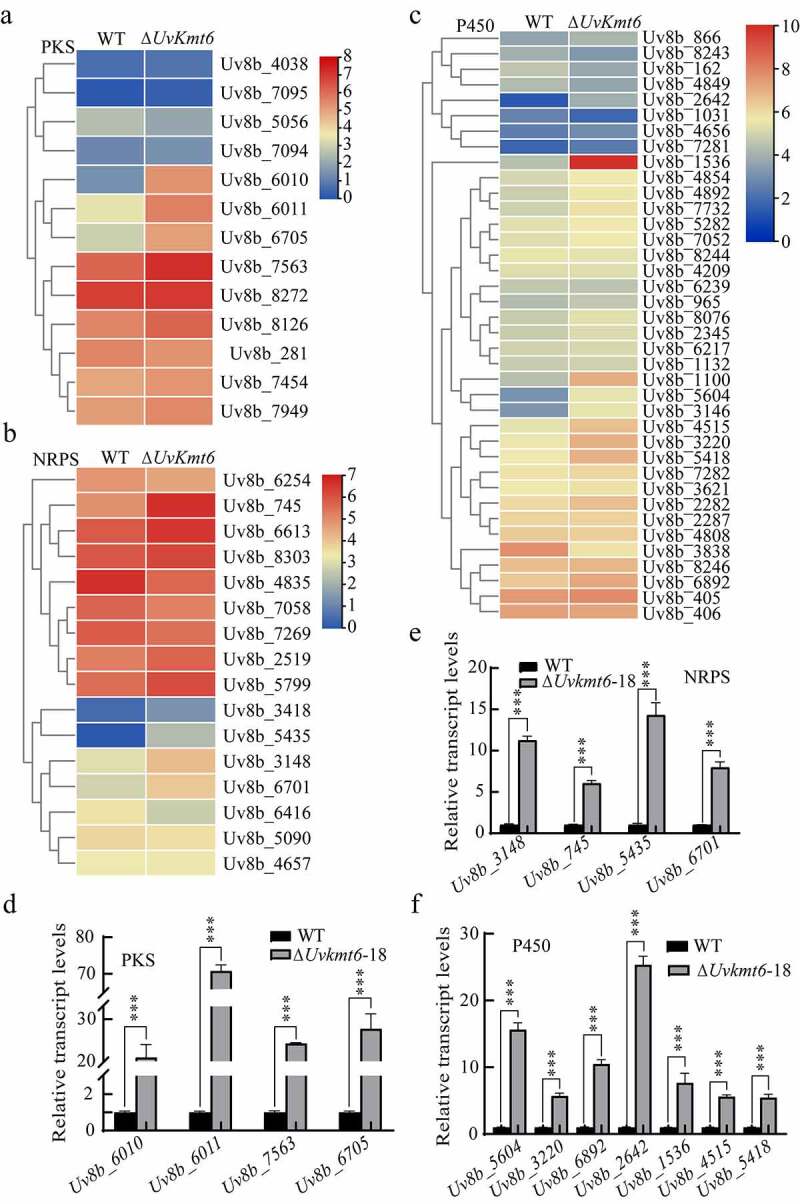
**Secondary metabolism-related genes *PKS, NRPS* and *Cytochrome P450*, are regulated by UvKmt6**. (a-c) Heatmaps displayed expression level changes of *PKS, NRPS* and *Cytochrome P450* in *U. virens*. Values coded by colors mean log_2_ of the ratio of FPKMs (Δ*Uvkmt6-*18/WT) and the scales were shown on the right. Total mRNA was extracted from mycelia of the WT and the Δ*Uvkmt6* strains cultured in PS media to verify the expression levels of 4 *PKS* (d), 4 *NRPS* (e) and 7 *Cytochrome P450* genes (f) by qRT-PCR. Bar represents standard error from three repeats. Asterisks mean significance at P ˂ 0.001 level

Genes encoding PKS (polyketide synthases), NRPS (nonribosomal peptide synthases), and Cytochrome P450 are involved in secondary metabolites synthesis and virulence in pathogenic fungi [22,26]. In *F. graminearum*, most of the genes encoding PKSs, NRPSs, and P450s are enriched with H3K27me3 modification, and are derepressed by deletion of *FgKMT6* [13]. To comprehensively understand the transcriptional regulation of secondary metabolites synthesis genes by H3K27me3 modification, expression levels of 14 *PKS*, 17 *NRPS* and 39 *Cytochrome P450* genes were compared in the WT and Δ*Uvkmt6* strains using the RNA-seq data. In contrast to WT, 11 of 14 *PKSs*, 9 out of 17 *NRPSs* and 20 of 39 *Cytochrome P450* genes were significantly up-regulated in the Δ*Uvkmt6* strain (Figure 7a-c). To verify RNA-seq results of these *PKSs, NRPSs* and *Cytochrome P450*s genes, qRT-PCR assays were carried out. Consistent with RNA-seq data, deletion of *UvKMT6* resulted in more than 6 folds up-regulation of 4 *PKS* genes including *Uv8b_6010, Uv8b_6011, Uv8b_7563* and *Uv8b_6705* (Figure 7d). When *UvKMT6* was disrupted, transcriptional levels of 4 *NRPS* genes including *Uv8b_3148, Uv8b_745, Uv8b_5435* and *Uv8b_6701* also increased 3–70 folds in the Δ*Uvkmt6* mutants compared with those in the WT strain (Figure 7e). In addition, expression levels of 7 selected *Cytochrome P450* genes including *Uv8b_5604, Uv8b_3220, Uv8b_6892, Uv8b_2642, Uv8b_1536, Uv8b_4515* and *Uv8b_5418* increased at various degrees from 6 to 25 folds (Figure 7f). Furthermore, ChIP-qPCR assay of the qRT-PCR-tested up-regulated genes showed that most of them (12/15) were occupied with H3K27me3 modification except genes *Uv8b_745, Uv8b_3220* and *Uv8b_4515* (Figure S4). These results suggested that UvKmt6 participates in regulation of various secondary metabolites-related genes by H3K27me3-mediated repression in *U. virens*.

The toxic compounds and/or metabolites produced by the *U. virens* cultured in liquid PS media could inhibit germination of rice seeds [31,32,34]. Due to the significantly decreased H3K27me3-occupancy and increased relative transcription level of many secondary metabolic genes when *UvKMT6* was deleted, it is suggested that the toxic compounds and/or metabolites produced by *U. virens* may be changed. Therefore, the toxicity of culture filtration of strains WT, Δ*Uvkmt6*-16, −18 and Δ*Uvkmt6*-C to rice seed germination was evaluated. The results showed that the length of shoots treated with filtration of Δ*Uvkmt6*-16 and −18 was significantly shorter than those of WT and Δ*Uvkmt6*-C strains (Figure S3), further indicating that disruption of *UvKMT6* led to secondary metabolic disorder.

### UvKMT6 *is involved in response to various stresses*

**Figure 8.**
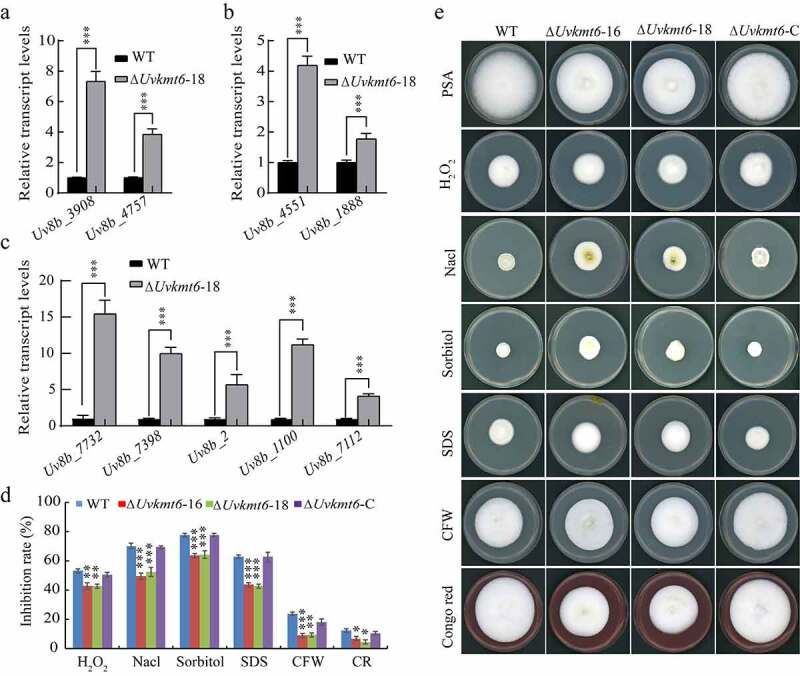
**UvKmt6-mediated H3K27me3 modification contributes to the response to various stresses**. The relative transcriptional levels of stresses response-related genes including cell wall stress-related genes (a), osmotic stress-related genes (b) and oxidative stress-related genes (c) were increased in the Δ*Uvkmt6-*18 mutant in comparison with WT. The relative transcriptional levels were estimated by comparing the expression levels of genes with that of the reference gene β-tubulin. Relative transcript abundances were calculated using the 2^−ΔΔCT^ method. (d) The Δ*Uvkmt6*-16 and 18 mutants were less sensitive to cell wall, osmotic and oxidative stresses. Mycelial plugs were inoculated on PSA plates supplemented with H_2_O_2_, NaCl, Sorbitol, SDS, CFW and Congo red for 14 d before photographed. (e) The bar chart showing the relative growth inhibition rate under various stress conditions. Values represent the mean ± SD from three independent repeats. *, ** or ***, P ˂ 0.01, P ˂ 0.005 or P ˂ 0.001

In order to survive and complete infection, *U. virens* fungus needs to adapt to various stresses from the host and environment [35]. By RNA-seq analysis, we found that a lot of stress response-related genes were affected extensively when *UvKMT6* was deleted (Table S3 and S4). Cell wall stress response-related genes, including *Uv8b_4757* (*chitin synthase 4*) and *Uv8b_3908* (*class 2 chitin synthase*), were confirmed to be up-regulated by qRT-PCR assay (Figure 8a). Similar transcriptional up-regulation was also found among osmotic stress response-related genes, including *Uv8b_1888* (*UvHOG1*) and *Uv8b_4551* (*UvSKN7*), and oxidative stress response-related genes, including *Uv8b_7732* (*mono-oxygenase), Uv8b_7398* (*ligninase h2 precursor), Uv8b_2* (*WSC domain containing protein), Uv8b_1100* (*cytochrome P450*) and *Uv8b_7112* (*laccase-like protein*) (Figure 8b and c). ChIP-qPCR assay of these genes showed that most of them (6/9) were occupied with H3K27me3 modification except genes *Uv8b_4757, Uv8b_1888* and *Uv8b_7732* (Figure S3) in the WT strain. To test if UvKmt6 plays a role in adaptation to stresses, the WT, ∆ *Uvkmt6*, and ∆ *Uvkmt6*-C strains were inoculated on the PSA medium and PSA plates amended with oxidative stress reagent H_2_O_2_, osmotic stress reagent NaCl or sorbitol, or cell wall stress reagents SDS (sodium dodecyl sulfate), CFW (calcofluor white) and CR (Congo red) for 15 d. Compared with WT or complemented strain Δ*Uvkmt6*-C, the colonies of the Δ*Uvkmt6*-16 and −18 strains were bigger and displayed less sensitivity to all these stress-mimicking reagents including H_2_O_2_, NaCl, sorbitol, SDS, CFW and CR (Figure 8d and e). Therefore, it was indicated that UvKmt6 regulated the response to oxidative, osmotic and cell wall stresses in *U. virens*.

**Figure 9.**
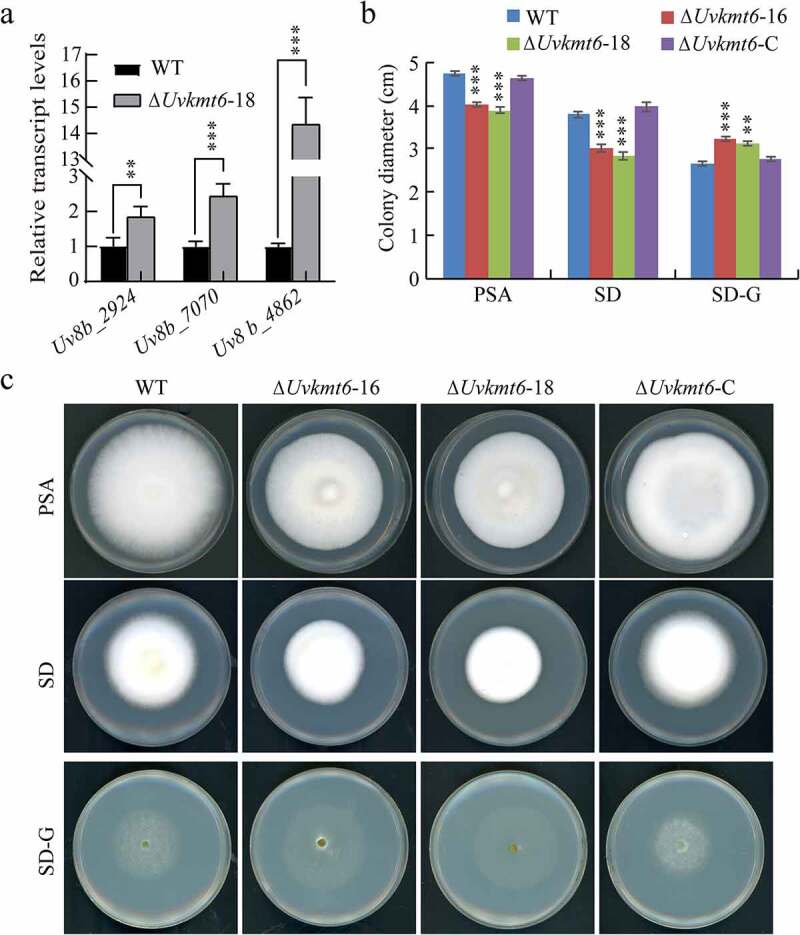
**The UvKmt6 is related to carbon sources starvation response**. (a) Expression levels of carbon utilization-related genes increased in the Δ*Uvkmt6-*18 strains were verified by qRT-PCR assay. (b and c) UvKmt6-mediated H3K27me3 modification plays a role in response to nutrient starvation stress. The WT, Δ*Uvkmt6* and Δ*Uvkmt6*-C strains were grown on the PSA, SD, and SD-G plates for 14 d, and then photographed. The colony of Δ*Uvkmt6* cultured on PSA and SD plates was smaller than WT, but bigger on the SD-G plates than WT. Data represents mean ± SD of three independent replicates. Asterisks highlight statistically significant difference (**, P ˂ 0.005; ***, P ˂ 0.001)

In addition, RNA-seq analysis also showed that genes related to nutrient utilization were regulated by UvKmt6 (Figure 5a, Table S3 and S4). qRT-PCR analysis further confirmed that the expression levels of genes related to carbon utilization, including *Uv8b_2924* (*putative beta-glucosidase), Uv8b_7070* (*glucan 1,3-beta-glucosidase*) and *Uv8b_4862* (*putative glucosidase*), were increased by 2 to 14 folds in the Δ*Uvkmt6* mutants (Figure 9a). Moreover, ChIP-qPCR assay of the three genes showed that both *Uv8b_2924* and *Uv8b_7070* were occupied with H3K27me3 in the WT (Figure S3), suggesting a regulatory role in response to nutrient condition by H3K27me3 modification in *U. virens*. Therefore, the growth of Δ*Uvkmt6*-16 and −18, WT and complemented strains were compared on PSA, SD (synthetic dropout medium) and SD-G (synthetic dropout medium without glucose) plates. When cultured for 7 d, the colony size of Δ*Uvkmt6*-16 and −18 mutants were smaller than that of WT or complemented strains on the PSA and SD plates. In contrast, the colony diameter of the Δ*Uvkmt6*-16 and Δ*Uvkmt6*-18 were larger than that of WT or complemented strains on SD-G plates (Figure 9b and c), suggesting that *UvKMT6* is involved in the response to nutrient stress.

## Discussion

Epigenetic modification of H3K27me3 established by PRC2 complex plays important roles in transcriptional regulation and development in various organisms [1]. *U. virens* caused rice false smut disease is a threat to rice production and food safety due to the formation of smut balls and mycotoxins [19]. In order to grasp the biological roles of PRC2-mediated H3K27me3 modification in *U. virens*, we investigated the functions of the uncharacterized homologue of methyltransferase Kmt6, which is a core component of PRC2 complex, in this study. We noted that, unlike essential roles of Kmt6 in *F. fujikuroi* [36], *UvKMT6* gene was knocked out successfully. Using the Δ*Uvkmt6* mutants, we found that the UvKmt6-mediated H3K27me3 modification plays significant roles in development and pathogenicity in *U. virens*. In addition, transcriptome profiling, ChIP-seq assay in combination with phenotypic analysis revealed that UvKmt6 participates in extensive regulation of a wide range of genes including *effectors*, secondary metabolites synthesis genes, and stress response-related genes.

The characterization of virulence-related factors will better help us to reveal the infection mechanism of *U. virens*. In this study, an epigenetic regulator UvKmt6 was found to contribute to the virulence of *U. virens* in several ways. First, disruption of *UvKMT6* resulted in drastic decrease in conidiation, which usually leads to reduced pathogenicity in *U. virens* [35]. For example, UvAtg8 is an autophagy marker, and its gene deletion mutant is significantly reduced in pathogenicity due to the decrease of conidia production [32]. Moreover, transcriptional factor UvCom1 and UvHox2 involved in conidiation are also necessary for the rice false smut balls formation [37,38]. The reduction in conidiation could be caused by the transcriptional dysregulation of conidiation-related genes in the deletion mutant of *UvKMT6* (Table S3 and S4). Second, approximately a quarter of putative *effectors* were abnormal expressed when *UvKMT6* was deleted. As we know, effectors secreted by pathogens are employed to suppress host immunity and contribute to virulence [28,29,39]. The fact that downregulation of *UvKMT6* during *U. virens* infection at 7 dpi also suggests that it contributes to releasing transcriptional suppression of H3K27me3-repressed genes such as *effectors* and promote infection. Therefore, we infer that the abnormal expression of effector proteins may be an important reason for the decrease of virulence in Δ*Uvkmt6* mutants. Third, the deletion of *UvKMT6* resulted in alteration of stress sensitivity in *U. virens*, which may also be one of the reasons for the reduced virulence of Δ*Uvkmt6* mutants. In previous reports, it has been implicated that alteration of stress sensitivity links to virulence of *U. virens*. For instance, absence of the cAMP signaling pathway component UvAc1 and UvPdeH or MAPK UvPmk1 displayed differential sensitivity to stress-mimicking reagents and reduced virulence [30,33]. As H3K27me3 is a hallmark of facultative heterochromatin regions of the genome, which has been reported to be associated with genome stability [11], it is very possible that deletion of UvKmt6 may also affect the genome stability in *U. virens*. In conclusion, UvKmt6 plays an important role in the virulence of *U. virens* likely by regulating the conidiation, maintaining spatial and temporal specific expression of *effectors*, genome integrity and responding to stresses.

Enrichment of H3K27me3 on chromosomes depends on the Kmt6 methyltransferase in PRC2 complex [11,13,16]. In *U. virens*, UvKmt6 is also essential for marking genic regions with H3K27me3, which is demonstrated by immunoblot and ChIP-seq (Figure 4). It was known that part of H3K27me3-marked genes tend to be silenced in different filamentous fungi, such as in *N. crassa, M. oryzae, F. graminearum* and *F. fujikuroi* [3,13,17,36]. Notably, genes involved in secondary metabolism in *F. graminearum* and *F. fujikuroi* are marked with H3K27me3, and disruption of *KMT6* also derepresses expression of *PKS, NRPS* and *Cytochrome P450* genes [13,36]. Here, we found that the transcriptional repression activity was also associated with local enrichment of H3K27 methylation in *U. virens*. This is supported by the fact that 140 genes of 321 H3K27me3-occupance genes were up-regulated in the *UvKMT6* deletion mutant, while only 12 down-regulated genes overlapped with 321 H3K27me3-occupance genes. Furthermore, disruption of *UvKMT6* induced expression of *effectors*, secondary metabolism-related genes including *PKS, NRPS* and *Cytochrome P450* genes and stress response-related genes, which were occupied by H3K27me3 modification in the WT. Thus, our results further implied that the roles of H3K27me3 in transcriptional repression are relatively conserved.

In addition to the relatively conservative regulation of *effectors* and secondary metabolic gene expression, we further found that UvKmt6 appears to be a negative factor in responding to cell wall stress, osmotic stress, and oxidative stress in *U. virens*. The increased tolerance to various stresses in the *UvKMT6* deletion mutant might be caused by UvKmt6-mediated repression of stress response-related genes such as chitin synthases-encoded genes *Uv8b_3908* and *Uv8b*_*4757*, osmotic stress responding genes *UvHOG1* and *UvSKN7*, and oxidative stress responding genes including *laccase* and *cytochrome P450*s. In *U. virens*, knockout of MAPK HOG1 encoding gene *UvHOG1* resulted in increased sensitivity to osmotic and cell wall stresses, and down-regulated transcriptional expression of stress response-related gene *UvSKN7* [31]. Therefore, the increased expression of *UvHOG1* and *UvSKN7* may be one of the explanations for the tolerance of Δ*Uvkmt6* mutants to osmotic and cell wall stresses. Except cell wall, osmotic and oxidative stresses, Δ*Uvkmt6* mutant also showed a better growth than WT strain under carbon source starvation stress. These phenotypes may be associated with increased expression of glucosidase-encoded genes in Δ*Uvkmt6* mutant. It has been reported that glucosidases catalyze efficient hydrolysis of β-glucan to produce glucose in *M. oryzae* [40]. The underlying mechanism of UvKmt6-mediated H3K27me3 modification on expression of these stress response-related genes need to be further studied.

Taken together, our results shed light on important roles of UvKmt6-mediated H3K27me3 modification in transcriptional repression of *effectors* during vegetative growth stage, secondary metabolites, stress response, development, and pathogenicity in *U. virens*. As far as we know, our study is the first report on depicting roles of epigenetic modification in *U. virens*, which extends current understanding of epigenetic regulation in pathogenic fungi and supports the link between H3K27me3 modification and infection-related biological processes in *U. virens*.

## Materials and methods

### Strains and growth condition

The *U. virens* WT strain HWD-2 was a kind gift from Prof. Junbin Huang of Huazhong Agriculture University (China). The strains were cultured on potato sucrose agar (PSA, potato 200 g/L, sucrose 20 g/L and agar 20 g/L) plates in constant dark chamber at 28°C. To evaluate vegetative growth, mycelial plugs were cut from 14-d-old PSA culture and transferred to new PSA plates for 14 d. To collect mycelia and conidia, mycelial plugs grew in liquid potato sucrose media shaking at 180 rpm for 7 d. Conidia were harvested from the filtrate by filtering the liquid cultures with three layers of gauze to determine conidial concentrations with a hemocytometer.

### The construction of vectors and transformation

To generate the deletion mutants of *UvKMT6*, a gene replacement strategy was used [32]. Briefly, approximate 1 Kb of 5ʹ UTR and 3ʹ UTR regions were amplified from the genomic DNA of the HWD-2 strain using the primer pairs UvKMT6-5 F/R and UvKMT6-3 F/R (Supplementary Table S1). Then, these flanking fragments were ligated sequentially to the flanking of *hygromycin resistance gene* cassette in the *pFGL821* (Addgene, 58,223). The sequence of resultant plasmid was confirmed by sequencing and subsequently introduced into the HWD-2 strain by ATMT. To generate the complementary vector, the *UvKMT6* fragment containing 1.5 Kb of promotor and coding region was amplified (primers listed in Table S1) and ligated into the vector *pFGL823* [34]. The sequence of plasmid was confirmed by sequencing, and then introduced into the deletion mutant by ATMT to obtain the complemented strains. All the correct transformants were confirmed by PCR, qRT-PCR and Southern blot assay (primers listed in Table S1) [32].

### Stresses treatments

To determine sensitivity to various stresses, mycelial plugs were inoculated on the PSA plates supplemented with 0.015% H_2_O_2_, 0.3 M NaCl, 0.5 M sorbitol, 0.03% SDS, 120 mg/mL CFW or 120 mg/mL CR, respectively. Stress sensitivity was assessed by measuring the diameter of the plates after cultured for 14 d at 28°C. The formula of relative inhibition rate was calculated as follow: Growth inhibition rate = (diameters of strain colony on the PSA – diameters of strain colony on the PSA amended with different chemicals)/diameters of the strain colony on the PSA × 100%. For the nutrient starvation assay, the SD (yeast nitrogen base without amino acids 1.7 g/L, ammonium sulfate 5 g/L, glucose 20 g/L and agar 20 g/L) medium and SD-G (yeast nitrogen base without amino acids 1.7 g/L, ammonium sulfate 5 g/L and agar 20 g/L) plates were used. Pictures were taken to present the vegetative growth under stress conditions. All these assays were repeated three times with at least three copies each time.

### Inoculation assay

The inoculation assay was performed as previously described [25,34,41]. Briefly, seeds of the rice cultivar Wanxian 98 (a susceptible rice cultivar, *Oryza sativa* L. *indica*), were cultivated in field for approximate three months and transferred to greenhouse until the booting stage. The WT, Δ*Uvkmt6* and complemented strain Δ*Uvkmt6*-C were cultured in PS (potato 200 g/L, sucrose 20 g/L) media for 7 d. Then, the resultant cultures were broken into mycelial pieces to make a mixed suspension of mycelia with conidia, and the mixtures were normalized by adjusting conidia concentration to 1 × 10^6^/mL. 2 mL of the mixture was injected into panicles and all rice plants were cultivated at the relative humidity of 95% and 22°C for 2 d in the dark following 28°C for 3 weeks. The disease symptoms were represented by the number of false smut balls and the photo of infected panicles. Toxicity assays with culture filtrates were performed as described using Wanxian 98 seeds (Zheng et al., 2016). All experiments in this part were repeated three times in each test.

### Western blotting

For detecting histone modification, 0.2 g mycelia were collected from 7 dpi in the liquid PS and ground in liquid nitrogen. The ground mycelial powders were suspended with buffer I (20 mM Tris pH 7.5, 20 mM KCl, 2 mM MgCl2, 25% Glycerol, 250 mM Sucrose, 0.1 mM PMSF (Phenylmethanesulfonyl fluoride), 5 mM beta-mercaptoethanol, 1× proteinase inhibitors). The resulting solutions were filtered through one layer of Miracloth (Millipore) and centrifuged. Then, the pellets were suspended with buffer II (50 mM Tris-HCl pH 7.4, 150 mM NaCl, 1 mM EDTA, 1% Triton100 and 1× protein inhibitor) to obtain total nucleus proteins. Total nucleus proteins were subject to 15% SDS-PAGE and transferred to PVDF membrane, which immunoblotted with antibodies including anti-H3 (Huabio, M1309-1), anti-H3K27me3 (Active motif, 39,155), anti-H3K4me3 (Abcam, ab1012), anti-H3K36me3 (Abcam, ab9050) antibody, respectively. The results of immunoblotting were captured with an imaging system using a chemiluminescence kit (Bio-Rad).

### Chromatin immunoprecipitation (ChIP) and sequencing

The ChIP experiments were performed according to previous methods with minor modification [42,43]. Briefly, 7-d cultured mycelia cultured in the PS media were cross-linked with 1% formaldehyde for 20 mins. After glycine solution (125 mM) was added to stop crosslink, mycelia was harvested with three layers of gauze, ground and suspended with the nuclei extracting buffer (10 mM Tris pH 8.0, 10 mM Sodium butyrate, 400 mM Sucrose, 0.1 mM PMSF, 5 mM β-mercaptoethanol and 1 × proteinase inhibitors). The resultant nuclei were then lysed with 1 mL nuclei lysis buffer (50 mM HEPES pH7.5, 150 mM NaCl, 1 mM EDTA, 10 mM Sodium butyrate, 0.1% deoxycholate, 1% Triton X-100, 0.1% SDS, 1 mM PMSF and 1× Roche protease inhibitor cocktail). The nuclear DNA solution was broken into DNA fragments whose lengths ranged from 200 to 500 bp using a sonication machine Diagenode Bioruptor (Figure S5). After pretreating with protein A beads (Thermofisher, 10001D), the supernatant was incubated with anti-H3K27me3 antibody (Active motif, 39,155) overnight at 4°C. Then another piece of protein A beads was added into the above reaction system to bind anti-H3K27me3 antibody and then washed three times. The immunoprecipitated DNA was recycled and used for ChIP-seq and ChIP-qPCR. For ChIP-seq assay, the recycled DNA was sequenced using a high-throughput sequencing platform Illumina Hiseq-PE150 by the Novogene Corporation (China). The resulting clean read pairs were mapped to the genome of the Uv8b strain with Bowtie2 (Version 2.2.8) [44]. The peak calling was performed with MACS2 (Version 2.1.1) [45], and visualized with the integrative genomics viewer [46]. The ChIP-seq assay was conducted with two biological repeats. To verify whether genes were occupied by H3K27me3, ChIP-qPCR assays with two independent repeats were conducted with gene *TUB* as internal reference. The qPCR assay was performed by using the immunoprecipitated DNA with anti-H3K27me3 antibody from the wild-type strain and Δ*Uvkmt6* as templates with the tubulin gene (Uv8b_900) as internal control. The statistical analysis was performed with *t* test. The PCR primers used in these experiments were listed in the Supplementary Figure S6 and Table S1.

### qRT-PCR and RNA sequencing

Total RNA was isolated from mycelia cultured for 7 d in liquid PS with three independent biological repeats using the TRIzol (Invitrogen) reagent. Subsequently, cDNA were synthesized with a reversely transcribed kit (TAKARA). The cDNA was subjected to qRT-PCR assay with SYBR Green qPCR Master Mix (TAKARA) using the *tubulin* gene (*Uv8b_900*) as internal control. The primers used in the experiments were listed and described in Supplementary Table S1.

RNA sequencing and comparison of differentially expressed genes of indicated strains was performed as previously described [47]. Briefly, purified RNA concentration was measured with Qubit RNA assay kit and the RNA integrity was determined by Bioanalyzer 2100 RNA-6000 Nano Kit (Agilent Technologies, CA, USA). A total amount of 3 μg RNA was used for RNA sample preparations. The mRNA-seq libraries were constructed using NEBNext UltraTM RNA Library Prep Kit (New England Biolabs, California, America) according to the manufacturer’s instructions followed by sequencing on an Illumina Hiseq X-Ten with Hiseq-PE150 strategy at Novogene. The resulting clean reads after removing adapter, low quality reads and reads containing ploy-N with fastp were mapped to the *U. virens* reference genome using Hisat2 v2.0.5. Then the featureCounts v1.6.4 were used to generate raw counts. The gene transcript abundances were normalized and differentially expressed genes analysis was analyzed by DESeq2 software. Only the genes’ different expression with log2|FC| > 1 and P-adjust values < 0.05 were considered significantly differential expressed. GO enrichment analysis was carried out under the Bonferroni-corrected P ≤ 0.05 compared with the whole-transcriptome background. GO functional enrichment analysis was accomplished on the website (https://www.omicshare.com/tools/). Reference genome and gene model annotation files were downloaded from genome website directly (https://ftp.ncbi.nlm.nih.gov/genomes/all/GCA/000/687/475/GCA_000687475.1_Assembly_for_version_1_of_the_Villosiclava_virens_genome/). Three biological replicates were performed for each strain/mutant. To determine the significance of the association between two sets of genes, P value was obtained by Fisher’s exact test using TBtools [48].

## Supplementary Material

Supplemental Material

## Data Availability

The data that support the findings of this study are available upon reasonable request.
