# Supplementary figures and images for: UvKmt6-mediated H3K27 trimethylation is required for development, pathogenicity, and stress response in Ustilaginoidea virens
Source: Virulence. 2021 Dec 11;12(1):2972–88. doi: 10.1080/21505594.2021.2008150 (PMC8667953; doi:10.1080/21505594.2021.2008150)

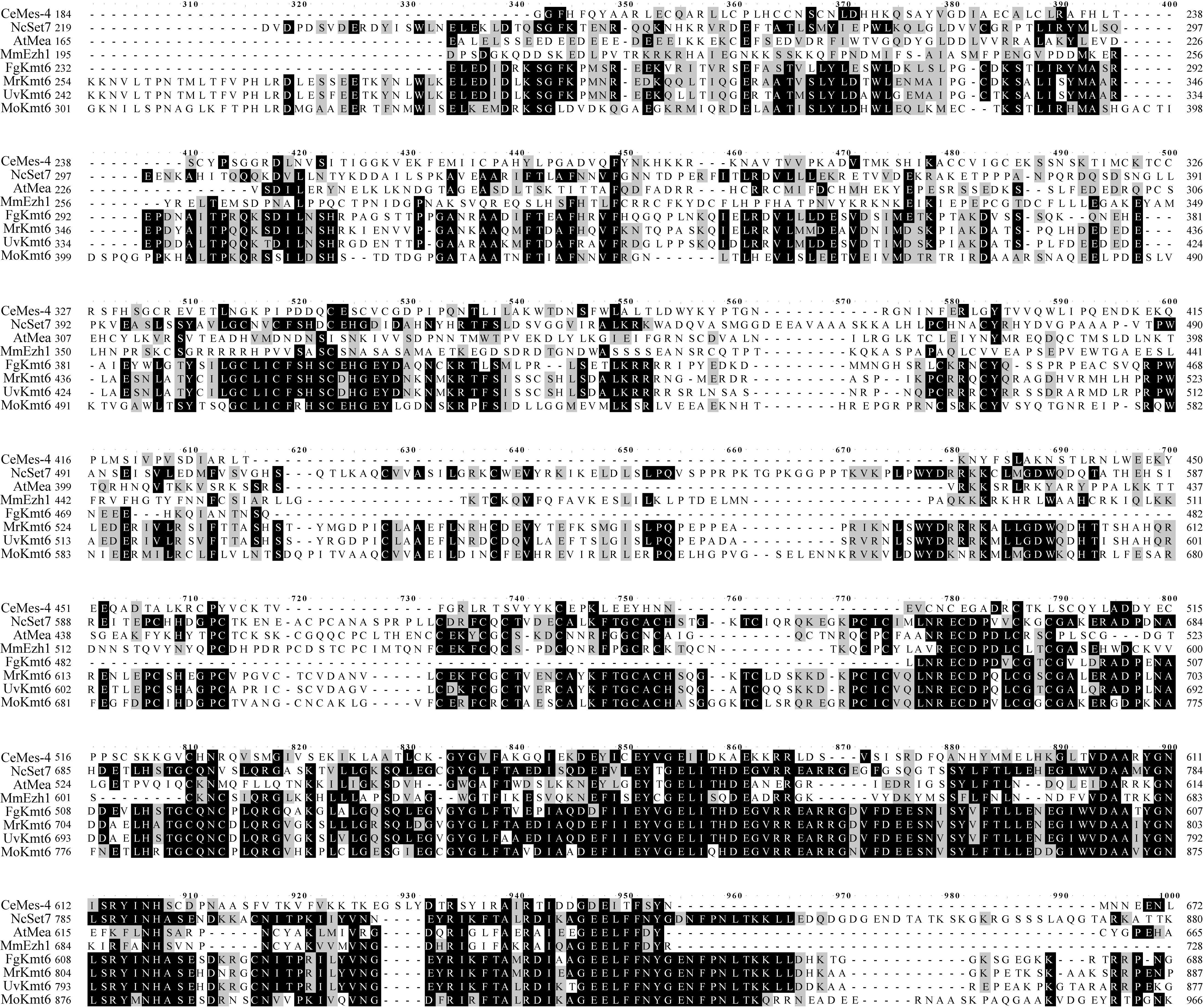

Supplement: Supplemental Material [file KVIR_A_2008150_SM1752.zip › supplementary/fig S1.tif]

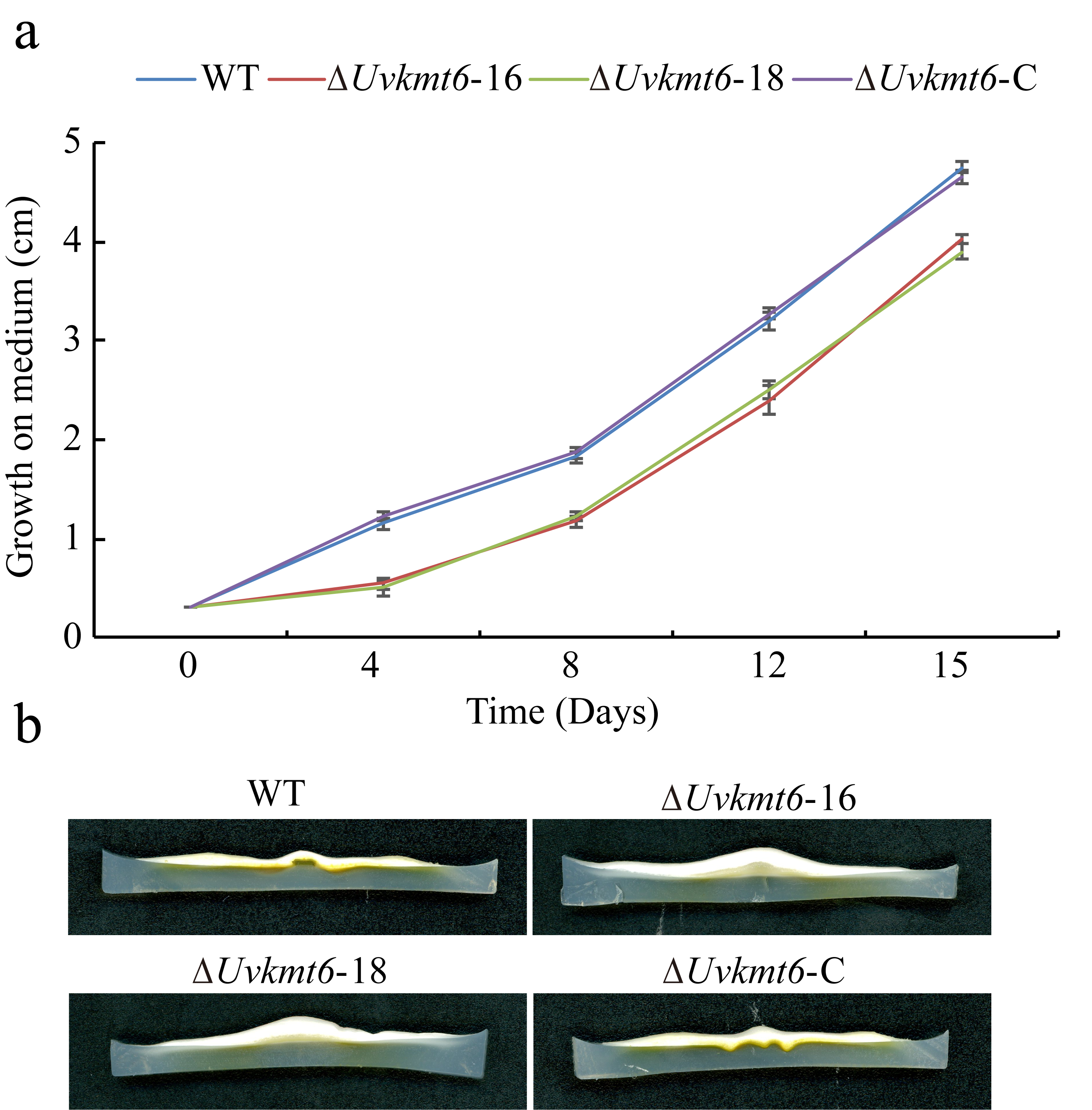

Supplement: Supplemental Material [file KVIR_A_2008150_SM1752.zip › supplementary/fig S2.tif]

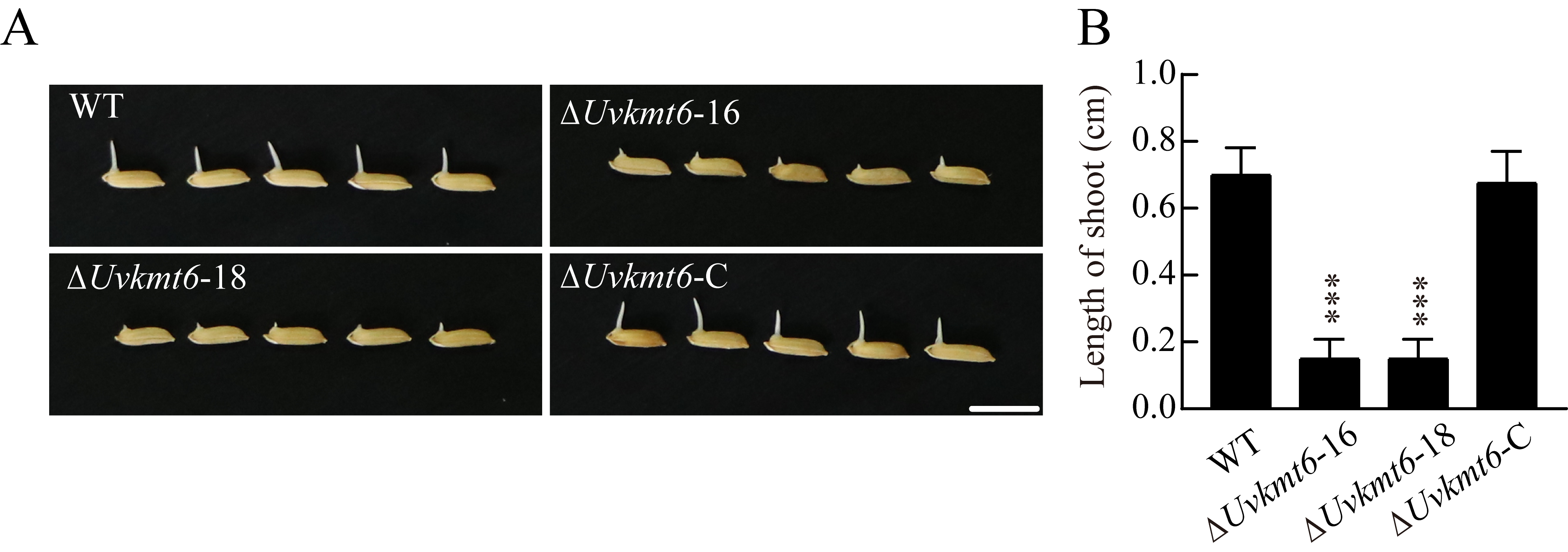

Supplement: Supplemental Material [file KVIR_A_2008150_SM1752.zip › supplementary/fig S3.tif]

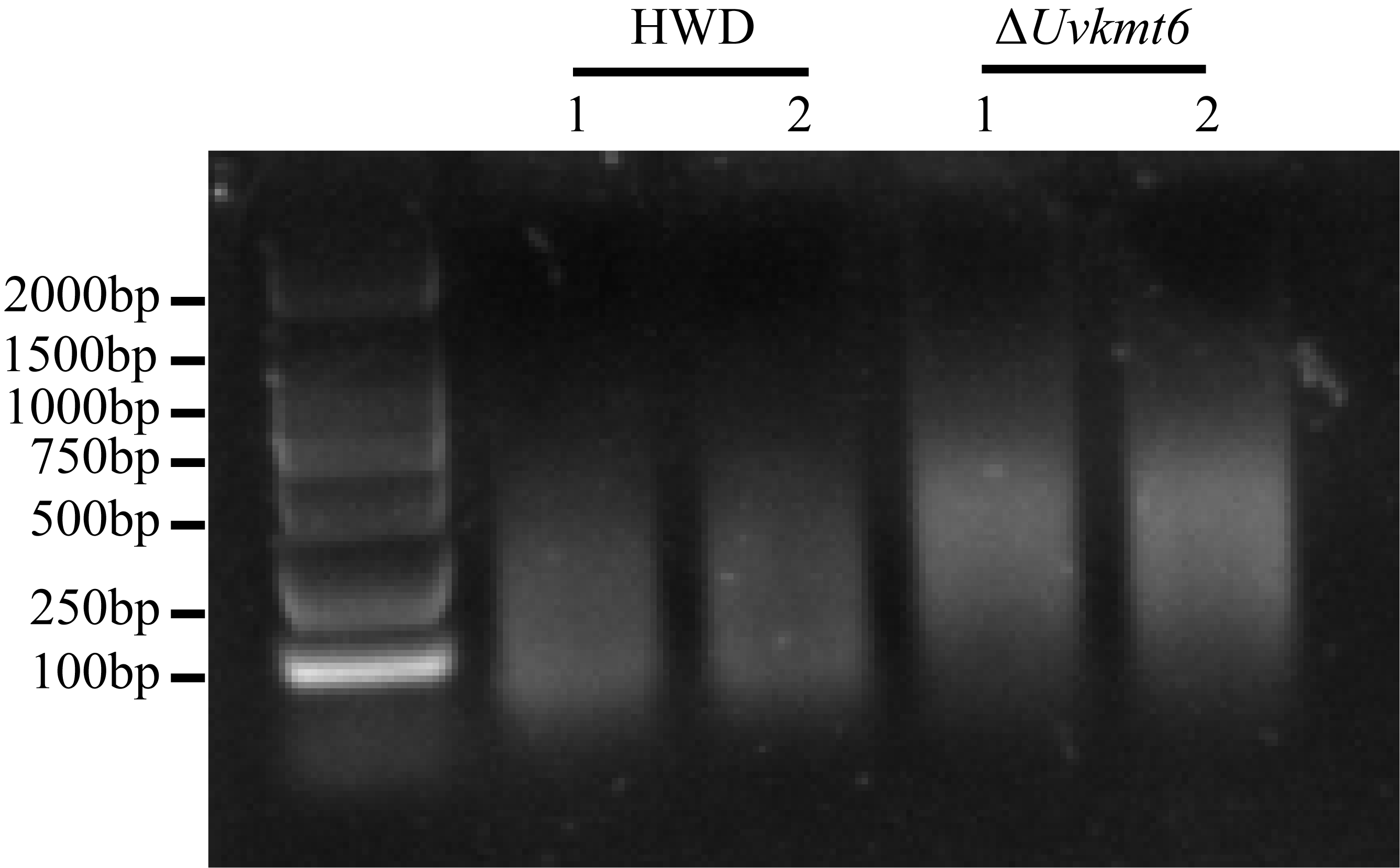

Supplement: Supplemental Material [file KVIR_A_2008150_SM1752.zip › supplementary/fig S5.tif]

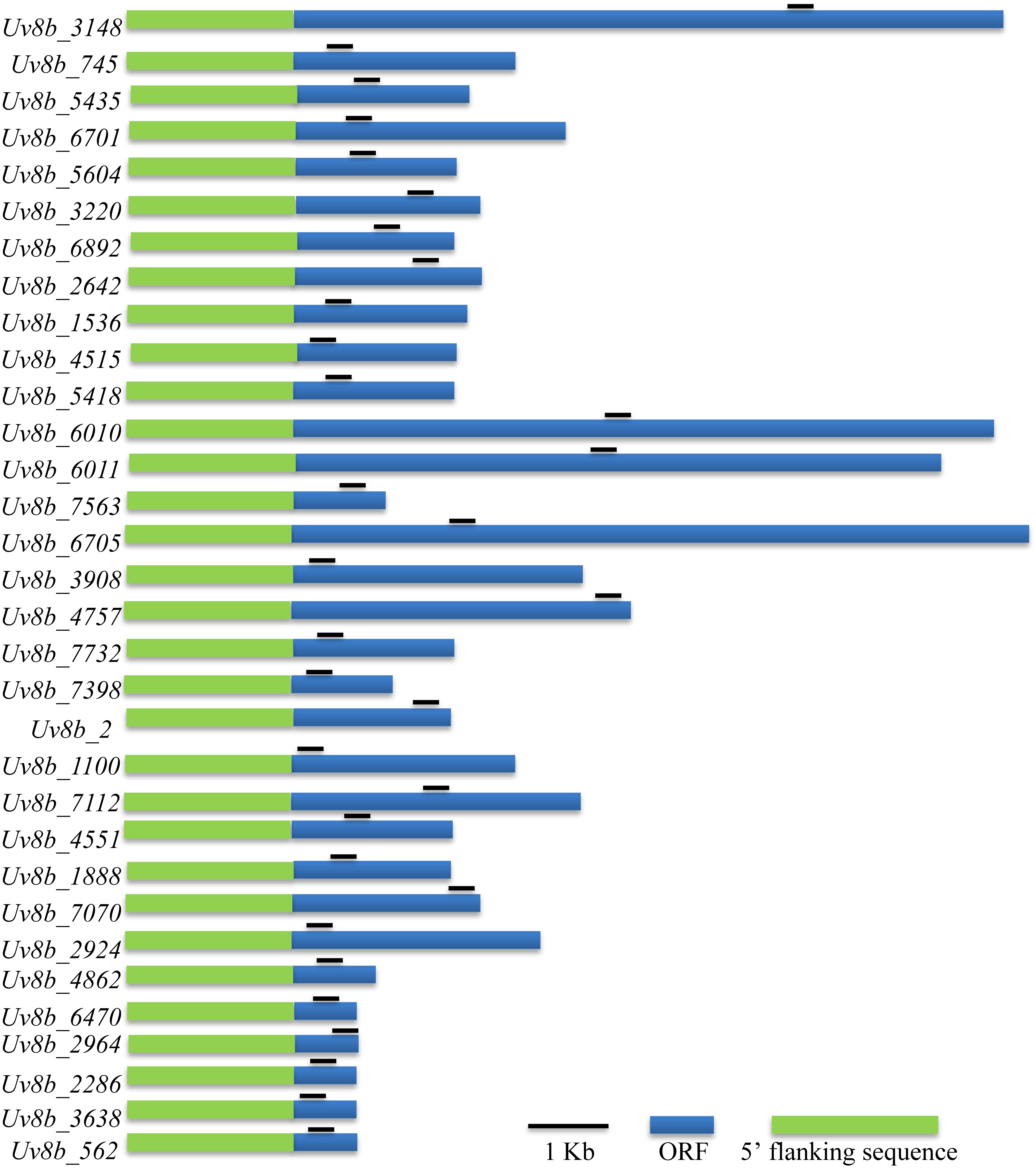

Supplement: Supplemental Material [file KVIR_A_2008150_SM1752.zip › supplementary/fig S6.tif]
